# Membrane targeting of TIRAP is negatively regulated by phosphorylation in its phosphoinositide-binding motif

**DOI:** 10.1038/srep43043

**Published:** 2017-02-22

**Authors:** Xiaolin Zhao, Wen Xiong, Shuyan Xiao, Tuo-Xian Tang, Jeffrey F. Ellena, Geoffrey S. Armstrong, Carla V. Finkielstein, Daniel G. S. Capelluto

**Affiliations:** 1 Protein Signaling Domains Laboratory, Department of Biological Sciences, Biocomplexity Institute, and Center for Soft Matter and Biological Physics, Virginia Tech, Blacksburg VA, 24061, USA; 2 School of Materials and Metallurgy, Inner Mongolia University of Science and Technology, P. R. China; 3 Biomolecular Magnetic Resonance Facility, University of Virginia, Charlottesville VA, 22904, USA; 4 Departments of Chemistry and Biochemistry, University of Colorado at Boulder, Boulder CO, 80309, USA; 5 Integrated Cellular Responses Laboratory, Department of Biological Sciences, Biocomplexity Institute, Virginia Tech, Blacksburg VA, 24061, USA

## Abstract

Pathogen-activated Toll-like receptors (TLRs), such as TLR2 and TLR4, dimerize and move laterally across the plasma membrane to phosphatidylinositol (4,5)-bisphosphate-enriched domains. At these sites, TLRs interact with the TIR domain-containing adaptor protein (TIRAP), triggering a signaling cascade that leads to innate immune responses. Membrane recruitment of TIRAP is mediated by its phosphoinositide (PI)-binding motif (PBM). We show that TIRAP PBM transitions from a disordered to a helical conformation in the presence of either zwitterionic micelles or monodispersed PIs. TIRAP PBM bound PIs through basic and nonpolar residues with high affinity, favoring a more ordered structure. TIRAP is phosphorylated at Thr28 within its PBM, which leads to its ubiquitination and degradation. We demonstrate that phosphorylation distorts the helical structure of TIRAP PBM, reducing PI interactions and cell membrane targeting. Our study provides the basis for TIRAP membrane insertion and the mechanism by which it is removed from membranes to avoid sustained innate immune responses.

The innate immune system is composed of germline-encoded receptor proteins that recognize invading pathogens by what is referred to as pathogen associated molecular patterns (PAMPs)^1^. Toll-like receptors (TLRs) are the best-studied group of innate immune receptors that are able to recognize various PAMPs, such as bacterial lipopolysaccharides (LPS) or double stranded RNA from viruses^2^. Ligand-activated TLRs self-associate, triggering the recruitment of cytoplasmic adaptor proteins. Through their TIR domains, TLR2 and TLR4 interact with the TIR domain-containing adaptor protein (TIRAP; also known as MAL) and the myeloid differentiation primary response gene 88 (MyD88) [reviewed in ref. 3]. TIRAP contains an N-terminal phosphoinositide (PI)-binding domain (PBD) followed by a TIR domain. Plasma membrane localization of TIRAP depends on the presence of phosphatidylinositol 4,5-bisphosphate (PtdIns(4,5)P_2_)-enriched regions^4^. TIRAP also serves as a bridge to recruit MyD88 through TIR-TIR domain interactions^5^. However, the presence of TIRAP at the plasma membrane is required even when there are low TLR ligand levels^6^. TIRAP’s association triggers further recruitment of members of the IRAK family of kinases to promote formation of the myddosome, which in turn activates TRAF6 and NF-κB nuclear translocation^7^. As a transcription factor, NF-κB mediates pro-inflammatory and anti-microbial gene expression. The structure of the TIRAP TIR domain reveals two potential dimerization interfaces in a configuration that allows the two monomers of the N-terminal PBD to be oriented in the same direction, facilitating PtdIns(4,5)P_2_-mediated plasma membrane targeting^8^,^9^.

Plasma membrane targeting of TIRAP is likely mediated by a short stretch of basic residues [amino acids 15–35; herein named PI-binding motif (PBM)] within the putative PBD^10^. Alanine mutagenesis of TIRAP residues Lys15, Lys16, Lys31, and Lys32 or hydrolysis of cellular PtdIns(4,5)P_2_ by bacterial phosphatases abrogates TIRAP’s plasma membrane targeting and reduces TIRAP’s PtdIns(4,5)P_2_ binding^10^. TIRAP chimeras, in which the PtdIns(4,5)P_2_-binding region is replaced by the PtdIns(4,5)P_2_-binding region of PLCδ1, still signals from LPS-induced TLR4^10^. More recently, TIRAP has been shown to be required for endosomal TLR9 signaling, which is triggered by viral ligands^11^. Due to the broad preference for acidic phospholipids, TIRAP is proposed to localize, in addition to the plasma membrane, to endosomes enriched with PtdIns(3)P to assist with the myddosome assembly in these compartments^11^.

Synthesis and turnover of plasma membrane PtdIns(4,5)P_2_ influences TIRAP subcellular localization. Membrane binding of TIRAP is regulated by phosphatidylinositol-5 kinase (PI5K), an enzyme that generates intracellular PtdIns(4,5)P_2_ and co-localizes with TIRAP at the plasma membrane^12^. Moreover, TIRAP interacts with PI3Ks, enzymes that convert PtdIns(4,5)P_2_ into PtdIns(3,4,5)P_3_, impairing TIRAP’s membrane targeting^13^. The activity of TIRAP upon TLR2 and TLR4 activation is tightly regulated by sequential events of phosphorylation and ubiquitination. TIRAP is phosphorylated by the Bruton’s tyrosine kinase (Btk) at the TIR domain, which is required for TIRAP signaling^14^. However, activation of TIRAP by phosphorylation is transient, as it later contributes to the rapid degradation of the protein shortly after TLR2 or TLR4 activation. Phosphorylation of TIRAP by Btk is required for subsequent polyubiquitination by the ubiquitin ligase suppressor of cytokine signaling 1 (SOCS1), leading to TIRAP degradation^15^. Thus, Btk-mediated degradation of TIRAP is proposed to avoid sustained TLR2 and TLR4 signaling and, consequently, to impair chronic inflammation^15^. Rapid turnover of TIRAP is also regulated by the action of the serine/threonine kinases IRAK1 and IRAK4, enzymes that are part of the myddosome. Phosphorylation occurs at Thr28, within PBM, which targets TIRAP for ubiquitination and degradation^16^.

Here, we employed structural, biochemical, and subcellular localization studies to elucidate the mechanism of TIRAP’s membrane binding and dissociation through its PBM. Our findings establish that TIRAP PBM is an unfolded module that switches to a helical structure in dodecylphosphocholine (DPC) micelles as well as in the presence of monodispersed PtdIns(4,5)P_2_ and PtdIns(3)P. We identified basic and nonpolar residues in TIRAP PBM involved in PI interaction and demonstrated that phosphorylation in the inositol ring as well as the presence of fatty acids are required for binding. The U-shaped backbone-based solution structure of TIRAP PBM in DPC micelles shows a long helix with the PI-interacting residues located at both ends of the helix. Using a synthetic TIRAP PBM peptide phosphorylated at Thr28 and a pseudophosphorylated variant, in which Thr28 was replaced with aspartate, we established that phosphorylation distorts the helical structure in PBM and reduces PI binding, consequently, impairing cell membrane binding of TIRAP. Thus, misfolding of the membrane-interacting region of TIRAP by phosphorylation is a prerequisite for its rapid turnover.

## Results

### TIRAP PBM is intrinsically disordered but becomes helical when it interacts with micelles and PIs

**Figure 1:**
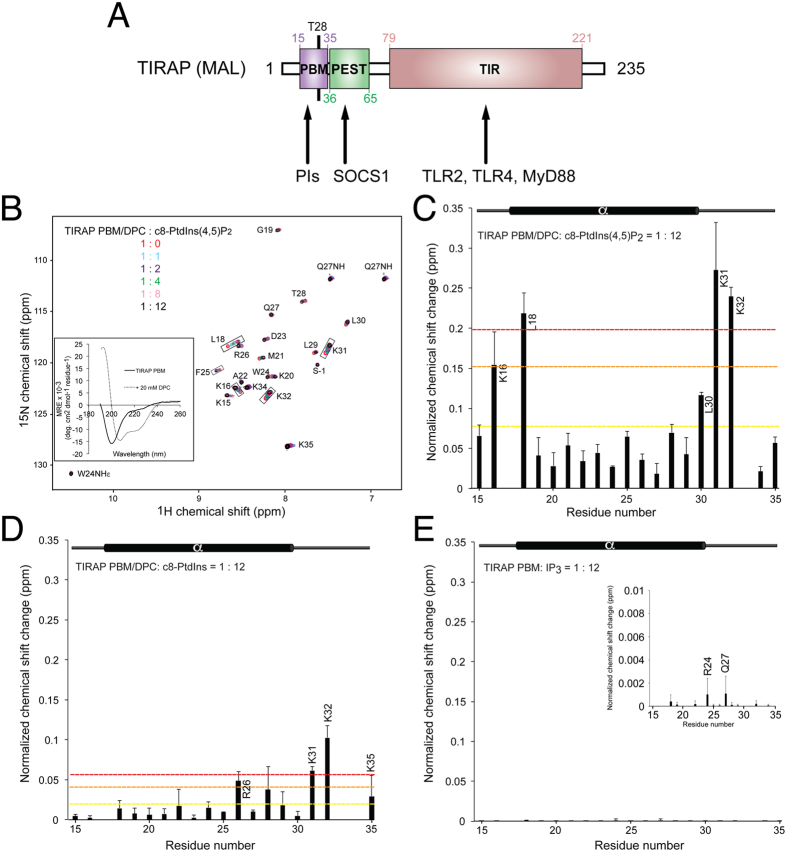
TIRAP PBM interacts with both head group and acyl chains of PIs. (**A**) Schematic representation of the topology of TIRAP including the protein and lipid-interaction sites. T28 is a site of phosphorylation within PBM. PEST, Pro-Glu-Ser-Thr-rich sequence; SOCS1, suppressor of cytokine signaling 1; TLR, Toll-like receptor; MyD88, myeloid differentiation primary response gene 88. (**B**) Overlay of ^1^H,^15^N-HSQC spectra of DPC-embedded TIRAP PBM in the absence and presence of the indicated molar ratios of c8-PtdIns(4,5)P_2_. The inset shows the far-UV CD spectrum of TIRAP in the absence and presence of DPC micelles. (**C**) Histogram representing the average ± standard deviation of chemical shift perturbations of TIRAP PBM upon DPC-embedded c8-PtdIns(4,5)P_2_ binding. Data analysis was obtained from two independent experiments. The colored dashed lines represent significant chemical shift changes: red (Δδ_average_ + 1.5 × standard deviation) >orange (Δδ_average_ + 1 × standard deviation) >yellow (Δδ_average_). Secondary structure of TIRAP PBM, reported in this article, is displayed at the top of the histogram. (**D**) Histogram representing the chemical shift perturbations of TIRAP PBM upon DPC-embedded PtdIns binding. (**E**) Histogram representing the chemical shift perturbations of TIRAP PBM upon the addition of IP_3_ in the presence of DPC micelles. Inset: a different scale for the normalized chemical shift changes of the same histogram is shown to better visualize the perturbations induced by IP_3_.

**Figure 2:**
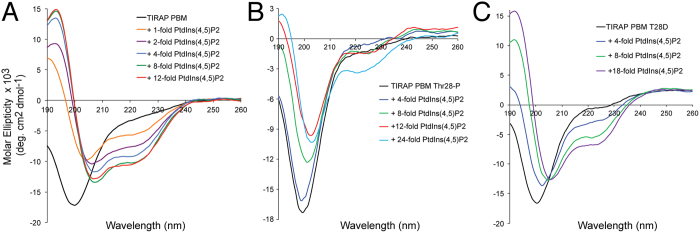
PI-induced folding of TIRAP PBM is impaired by phosphorylation in Thr28. Far-UV CD spectra of TIRAP PBM (**A**), TIRAP PBM Thr28-P (**B**), and TIRAP PBM T28D (**C**) in the absence and presence of the indicated molar ratios of c8-PtdIns(4,5)P_2_.

Previous studies identified TIRAP PBM (amino acids 15–35; Fig. 1A) to be sufficient and necessary for PI binding as well as for PtdIns(4,5)P_2_-dependent plasma membrane localization^10^. We found that the TIRAP PBM region was intrinsically disordered, but underwent a helical conformation in the presence of DPC micelles (Fig. 1B, inset). To obtain atomic-level details of the interaction of TIRAP with PtdIns(4,5)P_2_, the association of its PBM with the lipid was investigated by acquiring a series of ^15^N-^1^H HSQC spectra of DPC-embedded TIRAP PBM at different PtdIns(4,5)P_2_ concentrations (Fig. 1B). Specific chemical shift changes of conserved TIRAP PBM residues (Supplementary Fig. S1) were observed, including the N-terminal Lys16 and Leu18 residues as well as the C-terminal Leu30, Lys31, and Lys32 residues (Fig. 1B). Resonances of other basic residues in TIRAP PBM, including Lys15, Lys20, Arg26, Lys34, and Lys35, were not significantly perturbed (Fig. 1C), indicating that binding to the lipid was specific for a set of basic residues. Similar, but larger, resonance perturbations in DPC-embedded TIRAP PBM spectra were observed with the water insoluble c16-PtdIns(4,5)P_2_ (Supplementary Fig. S1), indicating that, during the NMR titrations, c8-PtdIns(4,5)P_2_ was incorporated into DPC micelles. PtdIns(3)P, which is reported to be the contact for TIRAP endosomal localization^11^, perturbed a similar set of PBM resonances but minor perturbations on Lys20 and Arg26 were also observed (Supplementary Fig. S1). Overall, these data suggest that the PtdIns(4,5)P_2_ and PtdIns(3)P binding sites overlap in TIRAP PBM. Binding of TIRAP PBM to phosphatidylinositol (PtdIns) was weaker (Fig. 1D), implying that the negative charges of the inositol phosphate groups contribute to peptide recognition. DPC-embedded TIRAP PBM did not interact with the head group of PtdIns(4,5)P_2_, inositol 1,4,5-trisphosphate (IP_3_) (Fig. 1E), indicating that interactions with the acyl chains are required for association to the lipid. The direct interaction of the disordered TIRAP PBM peptide with PIs was also investigated. HSQC spectra of TIRAP PBM at increasing concentrations of PtdIns(4,5)P_2_ displayed evident chemical shift perturbations suggesting that interaction is accompanied by a conformational change in the motif (Supplementary Fig. S2). Furthermore, TIRAP PBM adopted a helical conformation at monodispersed concentrations of PtdIns(4,5)P_2_ (Fig. 2A), PtdIns(3)P (Supplementary Fig. S2), and micellar concentrations of PtdIns (Supplementary Fig. S2). Interestingly, TIRAP PBM remained unfolded with IP_3_ at molar ratios as high as 20-fold (Supplementary Fig. S2). This is in agreement with the observation that the acyl chain is required for TIRAP PBM recognition (Fig. 1E). Overall, these data show that the charge in the lipid head group and the fatty acid tails are crucial for TIRAP PBM binding and folding.

**Figure 3:**
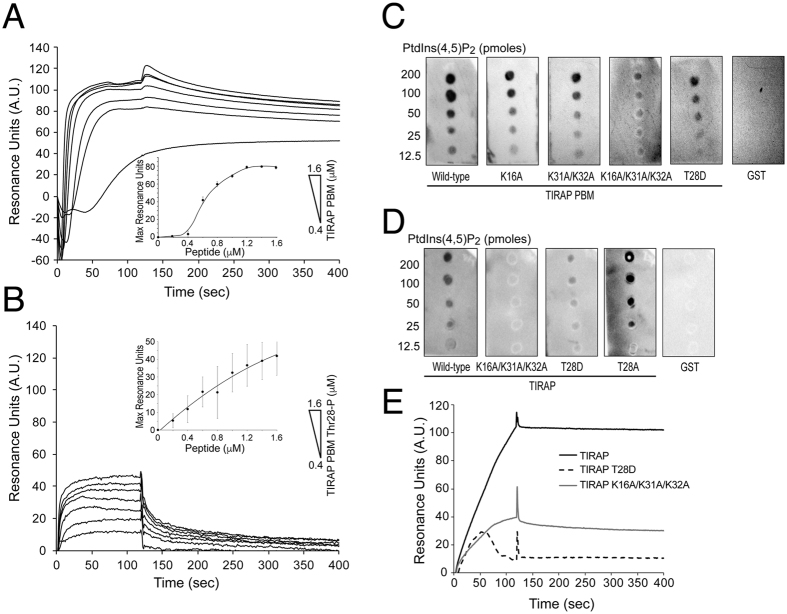
Binding of TIRAP to PtdIns(4,5)P_2_-enriched liposomes exhibits rapid on- and very slow off-rates and associations were reduced by PtdIns(4,5)P_2_-binding deficient mutants. Representative SPR traces flowing TIRAP PBM (**A**) or TIRAP PBM Thr28-P (**B**) over immobilized PtdIns(4,5)P_2_ liposomes. Insets, plot of the equilibrium response of each of the peptides for the estimation of their equilibrium *K*_D_ for PtdIns(4,5)P_2_. Each plot represents the average of three independent experiments. (**C,D**) Lipid-protein overlay assay of GST-TIRAP PBM (**C**) and GST-TIRAP (**D**) and the indicated mutants with immobilized c16-PtdIns(4,5)P_2._ GST was employed as a negative control. (**E**) Representative SPR traces flowing GST-TIRAP (black), GST-TIRAP K16A/K31A/K32A (gray), and GST-TIRAP T28D (black dashed) over immobilized PtdIns(4,5)P_2_ liposomes at 350 nM protein concentration. One representative experiment among two is shown.

DPC is commonly used as a membrane mimetic for solution NMR studies as it contains the head group of the most abundant phospholipid in eukaryotic membranes, phosphatidylcholine. However, micelles exhibit pronounced membrane curvature and form monolayers; consequently, protein-ligand affinity measurements in micellar environments may not be precisely estimated. Therefore, the interaction of TIRAP PBM with lipid bilayers enriched with PtdIns(4,5)P_2_ was quantified using surface plasmon resonance (SPR). Binding of TIRAP PBM to PtdIns(4,5)P_2_ liposomes exhibited a sigmoidal response with rapid association and very low dissociation phases (Fig. 3A). Kinetic analysis of these traces did not fit any of the kinetic models available (data not shown). Instead, we measured the equilibrium dissociation constant by plotting the steady state response units as a function of the concentration of TIRAP PBM (Fig. 3A, inset), resulting in an apparent dissociation constant (*K*_D_) of 233 ± 45 nM for PtdIns(4,5)P_2_-embedded liposomes.

Based on the PI-induced chemical shift changes in TIRAP PBM spectra, we replaced key-interacting residues in the peptide and evaluated their role in PI recognition using the lipid-protein overlay assay. Alanine mutations in Lys16, Lys31, and Lys32 reduced PtdIns(4,5)P_2_ and PtdIns(3)P binding (Fig. 3C and Supplementary Fig. S3), but did not affect the secondary structure of the peptide in DPC micelles (Supplementary Fig. S3). The effect of all these mutations could also be reproduced in full-length TIRAP (Fig. 3D and Supplementary Fig. S3). Using SPR analysis, we also measured binding of TIRAP to PtdIns(4,5)P_2_ liposomes. The interaction exhibited a rapid association phase without the presence of a steady state phase, followed by a very slow dissociation phase, suggesting a very high affinity interaction. TIRAP traces typically were ~4-fold higher than that for TIRAP K16A/K31A/K32A (Fig. 3E).

### TIRAP PBM exhibits a U-shaped structure with a long central helix

**Figure 4:**
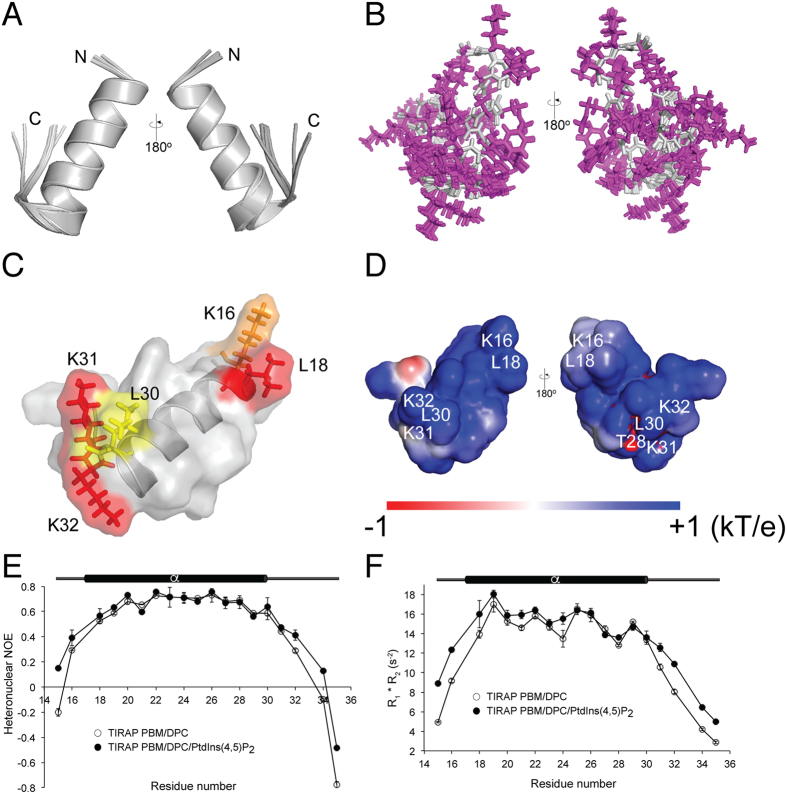
Solution structure and dynamics of TIRAP PBM in DPC micelles. Backbone ribbon trace (**A**) and all-atom view (**B**) of the overlaid twenty lowest-energy conformers of TIRAP PBM in DPC micelles. Side chain atoms are colored in purple, whereas backbone atoms are colored in gray. (**C**) Surface and ribbon representation of TIRAP PBM with the side chains of the residues involved in PtdIns(4,5)P_2_ binding. (**D**) Color-coded Van der Waals surface of TIRAP PBM in DPC micelles based on the electrostatic potential at the surface. (**E,F**) Backbone dynamics of DPC-embedded TIRAP PBM in the absence and presence of c16-PtdIns(4,5)P_2_. ^15^N, ^1^H NOEs (**E**) and R1 * R2 (**F**) relaxation parameters of individual residues of DPC-embedded TIRAP PBM were measured in the absence (empty circles) or presence (filled circles) of 12-fold excess of c16-PtdIns(4,5)P_2_. Each plot represents the average of two independent experiments.

The backbone-based structure of TIRAP PBM in the presence of DPC micelles was solved using the chemical shift assignments and refined using Rosetta. The overlay of the twenty lowest energy structures of TIRAP PBM in DPC micelles shows a central helix spanning residues Pro17 to Leu30 and flexible N- and C-termini (Fig. 4A,B). Overall, the structure of the peptide is well-defined as reflected by the average pairwise root mean square deviation for the backbone (0.3 Å) and heavy (0.6 Å) atoms (Table S1). The presence of a long helix in TIRAP PBM is in close agreement with the predicted secondary structure using JPred4 and TALOS-N (Supplementary Fig. S4)^17^. The DPC-bound structure of TIRAP PBM is characterized by a “U” shaped helical structure with the PI-interacting residues located at the helix boundaries (Fig. 4C). Based on the backbone-based structure of TIRAP PBM, the side chains of these residues face in the same direction (Fig. 4C), supporting their role in PI binding. A potential salt bridge in the TIRAP PBM helix, involving residues Asp23 and Arg26 (Supplementary Fig. S4), may be formed to tolerate the energetically unfavorable hydrophobic environment of the membrane. An image of the electrostatic potential on the solvent accessibility of TIRAP PBM, as estimated using Adaptive Poisson-Boltzmann Solver software^18^, displays an overall basic nature of the peptide, with the highest positive electrostatic potential delimited around the PI-interacting residues (Fig. 4D).

### Relaxation analysis of PtdIns(4,5)P_2_-bound TIRAP PBM reveals reduced mobility in the PtdIns(4,5)P_2_-binding residues

To investigate the dynamic properties of the micelle-bound TIRAP PBM in the absence and presence of PIs, we collected the heteronuclear ^15^N, ^1^H NOEs as well as the longitudinal (R_1_) and transverse (R_2_) relaxation rates. Data indicate that the DPC-bound TIRAP PBM region comprising Leu18 to Leu30 are located in a more rigid region (Fig. 4E,F) that overlaps with the central helix of the peptide. The rigidity of this region was not altered upon PtdIns(4,5)P_2_ binding (Fig. 4E,F). On the other hand, the N- and C-termini of DPC-embedded TIRAP PBM were more flexible and underwent an increment of restriction of flexibility upon PtdIns(4,5)P_2_ binding (Fig. 4E,F). Thus, the PtdIns(4,5)P_2_-dependent increment of order at the N- and C-termini of TIRAP PBM correlates with the location of the PtdIns(4,5)P_2_-interacting residues in the motif.

### Paramagnetic relaxation enhancement analyses demonstrate the depth of insertion of TIRAP PBM

**Figure 5:**
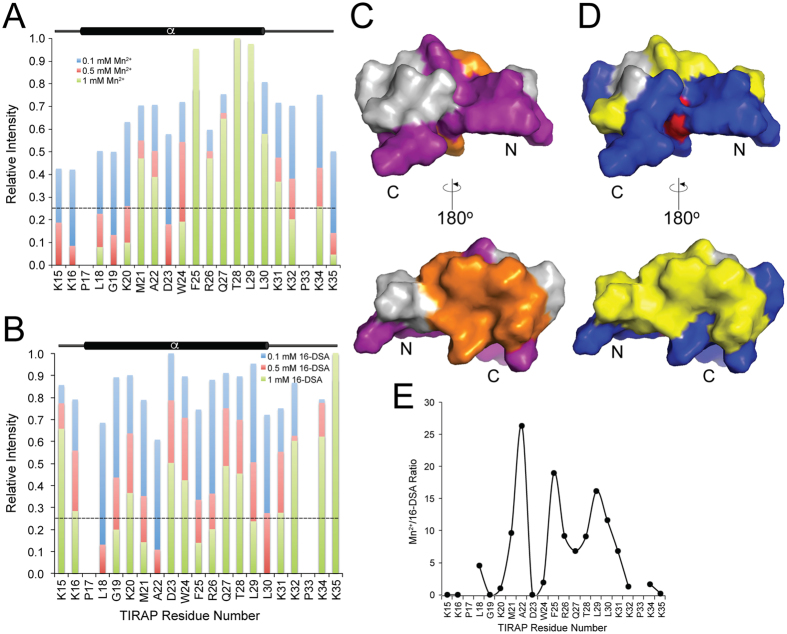
Probing the insertion of TIRAP PBM in DPC micelles. Paramagnetic relaxation enhancement of the backbone amide groups of TIRAP PBM induced by Mn^2+^ (**A**) and 16-DSA (**B**) at the indicated concentrations. (**C**) Two views of the surface representation of TIRAP PBM with the residues showing at least 75% reduction in signal by Mn^2+^ colored in purple (solvent-exposed) and by 16-DSA colored in orange (micelle-embedded). (**D**) Two views of the surface representation of TIRAP PBM color-coded according to the properties of its amino acids. Blue, positively charged; red, negatively charged; yellow, hydrophobic. (**E**) Mn^2+^/16-DSA-dependent paramagnetic relaxation enhancement ratio of TIRAP PBM resonance intensities.

To determine the depth of insertion of TIRAP PBM in DPC micelles, we performed paramagnetic relaxation enhancement experiments using two paramagnetic agents, Mn^2+^ and 16-doxyl stearic acid (16-DSA). Mn^2+^ enhances relaxation in residues that are solvent-exposed, but affects residues that are buried in the detergent micelle to a lesser extent. On the other hand, the doxyl moiety in 16-DSA is attached to the end of the aliphatic chain and, therefore, quenches cross-peaks of the peptide located at the core of the micelle. The N- and C-termini of TIRAP PBM were mostly affected by the Mn^2+^ ions (Fig. 5A), including PI-interacting residues such as Lys16, Lys31, and Lys32. Thus, membrane surface location of these residues is in agreement with the conformation of TIRAP PBM (Fig. 4C). On the other hand, most of the amino acids located in the helical region of the peptide strongly interacted with the doxyl group close the micellar core (Fig. 5B). Thus, a representation of the surface structure of TIRAP PBM displayed a clear partitioning of the solvent-exposed (colored in purple) and the micelle-embedded residues (orange; Fig. 5C). Interestingly, distribution of solvent-exposed TIRAP PBM residues mapped closely with the charged residues (blue), whereas the micelle-embedded residues were predominantly represented by hydrophobic residues (yellow; Fig. 5D). By obtaining the ratio of Mn^2+/^16-DSA of resonance intensities, the helix of TIRAP PBM shows evidence of an amphipathic nature with residues Ala22, Phe25, and Leu29 found deeply buried in the micelle, whereas Gly19, Lys20, Asp23, and Gln27 (to a lesser extent) located close to the micellar surface (Fig. 5E). The wheel diagram of the helix of TIRAP PBM (Supplementary Fig. S4) and the relative hydrophobic moment value of 0.456 supports the amphipathic nature of the peptide. In addition, the calculated discriminant factor value of 0.76 predicts that the helix of TIRAP PBM is possibly a lipid-binding region^19^. The addition of either c8-PtdIns(4,5)P_2_ or c16-PtdIns(4,5)P_2_ to DPC micelles did not cause any changes in the NMR peak intensities of TIRAP PBM (Supplementary Fig. S5), revealing that the lipid did not promote changes in peptide micellar insertion. Furthermore, substitution of c8-PtdIns(4,5)P_2_ by c16-PtdIns(4,5)P_2_ was indistinguishable (Supplementary Fig. S5), indicating that the length of the acyl chain of the lipid did not influence TIRAP PBM micellar insertion.

### Phosphorylation of TIRAP in Thr28 distorts the helical conformation in PBM

TIRAP is phosphorylated on Thr28 by IRAK proteins, leading to its ubiquitination and degradation^16^. Consequently, a Thr28-phosphorylated peptide representing TIRAP PBM (herein named TIRAP PBM Thr28-P) was synthetically produced (Supplementary Fig. S6). In contrast to the nonphosphorylated TIRAP PBM, PtdIns(4,5)P_2_ was unable to induce a helical structure in TIRAP PBM Thr28-P (Fig. 2B) and bound PtdIns(4,5)P_2_–enriched liposomes with a *K*_D_ of 787 ± 291 nM, which was ~3-fold weaker than TIRAP PBM (Fig. 3A,B). However, binding did not reach saturation and exhibited a much lower dissociation phase than TIRAP PBM (Fig. 3A), suggesting that the affinity value differences are higher than 3-fold. Since Thr28 was not identified as a residue critical for PI binding, it is possible that phosphorylation in the residue instead alters the helical structure of TIRAP PBM. Indeed, CD analysis showed that, as opposed to that observed for TIRAP PBM, DPC-embedded TIRAP PBM Thr28-P exhibited a poor secondary structure content as evidenced by the presence of a minimum at ~202 nm (Supplementary Fig. S3). This data can be explained by the fact that Thr28 is at the end of the helix in TIRAP PBM (Supplementary Fig. S4) and is away from the membrane surface independent of the presence of PtdIns(4,5)P_2_ as deduced from paramagnetic studies (Fig. 5 and Supplementary Fig. S5). The site-directed mutagenesis approach is routinely employed to genetically encode aspartate or glutamate as phosphomimics. Replacement of Thr28 by aspartic acid (TIRAP PBM T28D) closely mimicked the structure of TIRAP PBM Thr28-P in DPC micelles (Supplementary Fig. S3), impairing PtdIns(4,5)P_2_-induced folding (Fig. 2C) and DPC-induced folding (Supplementary Fig. S3). Furthermore, not only TIRAP PBM T28D but also TIRAP T28D bound PtdIns(4,5)P_2_ (Fig. 3D,E) and PtdIns(3)P (Supplementary Fig. S3) poorly, which is consistent with structure-dependent loss of function. Furthermore, SPR data showed a reduction in the binding of TIRAP T28D to PtdIns(4,5)P_2_-enriched liposomes, comparable to that observed for the TIRAP PtdIns(4,5)P_2_-deficient binding mutant (Fig. 3E). Interestingly, we observed that TIRAP T28D initially bound and then released from the sensorchip surface during the injection period (Fig. 3E). On the other hand, replacement of Thr28 to alanine (TIRAP T28A), as observed in mouse and rat TIRAP (Supplementary Fig. S1), did not alter PI binding (Fig. 3E) or the structure of PBM (Supplementary Fig. S3).

### Phosphomimic precludes TIRAP membrane localization

**Figure 6:**
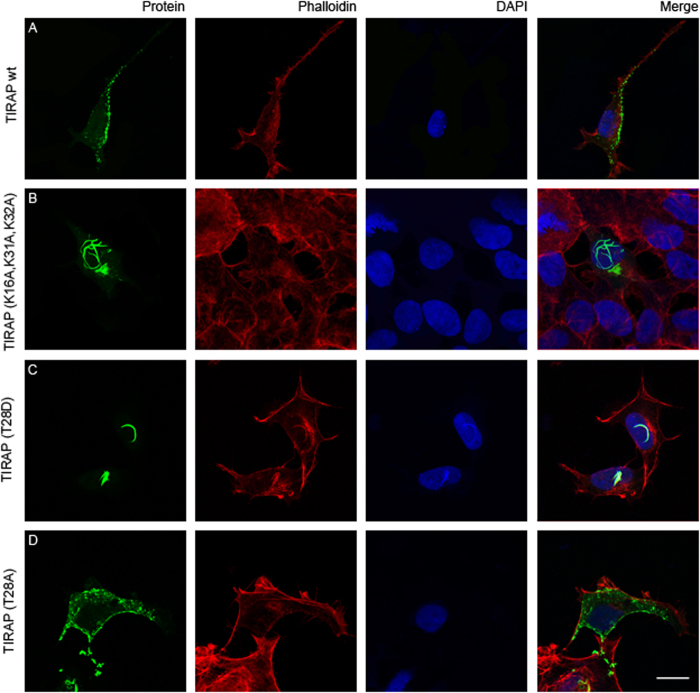
Mutations in either PtdIns(4,5)P_2_-binding residues or mimicking phosphorylation in PBM impairs TIRAP membrane binding. HEK293 cells were transiently transfected with plasmids expressing EGFP-tagged TIRAP (**A**), TIRAP (K16A,K31A,K32A) (**B**), TIRAP (T28D) (**C**), or TIRAP (T28A) (**D**) and proteins detected using immunofluorescence microscopy as described in the Experimental Procedures section. Rhodamine phalloidin and DAPI were employed as actin stress fibers and nuclei markers, respectively (Bar, 20 μm).

TIRAP typically localized in PtdIns(4,5)P_2_-enriched domains at the plasma membrane and in intracellular vesicle-like structures (Fig. 6A and Supplementary Fig. S7), consistent with published observations^10^,^20^. Alanine mutations in the PtdIns(4,5)P_2_-interacting residues (Lys16, Lys31, and Lys32) were sufficient to impair TIRAP plasma membrane binding (Fig. 6B). Interestingly, the phosphomimic TIRAP mutant, T28D, was also unable to localize to the plasma membrane and, instead, exhibited co-localization with fiber-like phalloidin-bound actin structures, on or near the nucleus (Fig. 6C and Supplementary Fig. S7), suggesting that this distribution could be a result of protein misfolding. However, future studies, which are beyond the scope of this report, are required to elucidate where these proteins are intracellularly retained. On the other hand, the TIRAP T28A mutant retained its plasma membrane-binding properties and its subcellular distribution was indistinguishable with that of the wild-type protein (Fig. 6D). Altogether, data suggest that PBM misfolding by phosphorylation in Thr28 is a prerequisite for the observed TIRAP ubiquitination and degradation.

## Discussion

In this article, we demonstrate that the PBM of TIRAP is a disordered module that adopts a helical conformation by interaction with PIs and membrane mimics. Flexible regions located at each end of PBM are responsible for direct contact with the PI, whereas the central helix is involved in membrane insertion of TIRAP. Interestingly, TIRAP PBM requires both the head group and the acyl chains of the lipid for interaction. Thr28 of TIRAP, which is phosphorylated by IRAK1 and IRAK4^16^ and is located at the end of the helix in PBM, is close to the micelle core. We show evidence that phosphorylation of Thr28 distorts the helical structure of TIRAP PBM, perhaps a prerequisite for its ubiquitination and degradation^16^. Through the combined use of a phosphopeptide (TIRAP PBM Thr28-P) and a TIRAP phosphomimic (TIRAP T28D), our data suggest that phosphorylation of PBM dramatically reduces binding of TIRAP to PIs and cell membrane binding. Thus, this mechanism can expose residues Lys15 and Lys16 for TIRAP ubiquitination^15^, abolishing the interaction with PIs and, consequently, reducing downstream signaling. Indeed, association of TIRAP to membranes is transient during TLR-mediated signaling. LPS-mediated activation of TLRs induces dynamic changes in PtdIns(4,5)P_2_ and TIRAP translocation to the plasma membrane, where TIRAP is transiently located at the plasma membrane and moves to the cytosol 30 min after LPS stimulation^12^.

TIRAP meets the general features of a peripheral membrane protein. First, membrane localization of TIRAP appears to be established in steady state by electrostatic interactions with PIs. This association is driven by its polybasic PBM region, which acts as a classical sensor of membrane charge provided by the enrichment of the lipid^21^. Second, as demonstrated in many other PI-binding modules, TIRAP PBM is promiscuous for lipid recognition^11^, with two interacting regions located at the ends of the motif that likely lay close to the surface of the cytosolic leaflet of cell membranes. Due to proximity, a potential salt bridge can be formed between the helical residues Asp23 and Arg26. Indeed, transmembrane proteins contain one or more salt bridges that allow them to reversibly insert into membranes and to compensate for the unfavorable hydrophobic environment of their charged residues^22^.

The PBM region of TIRAP has previously been demonstrated to be responsible for its binding to membranes by association to PIs^10^. TIRAP exhibits broad specificity for acidic phospholipids, with preference for PtdIns(4,5)P_2_ and PtdIns(3)P^11^. Alanine mutations of the TIRAP residues Lys15, Lys16, Lys31, and Lys32 severely reduced PI binding^10^. However, our biophysical data shed light into the molecular details of TIRAP’s PI association. NMR studies precisely pinpointed the critical PI-binding residues in TIRAP PBM through the use of membrane mimics. Whereas Lys16, Lys31, and Lys32 are critical for PtdIns(4,5)P_2_ binding, additional contacts to hydrophobic residues (Leu18 and Leu30) in TIRAP were also identified. Thus, this data supports the requirement of hydrophobic contacts, such as the acyl chains of the lipid, for binding. TIRAP PBM binding to PtdIns(4,5)P_2_-enriched liposomes was with nanomolar affinity and displayed sigmoidal kinetics, resembling the binding traces reported for TIRAP^13^. Similar sets of NMR resonance perturbations of TIRAP PBM were also observed in the presence of PtdIns(3)P, emphasizing the promiscuity of the PI binding site of TIRAP.

PtdIns(4,5)P_2_ is the most abundant PI in mammalian cells, representing 2–5% of the total lipid. PtdIns(4,5)P_2_ is mainly found at the plasma membrane but small amounts have been identified in the Golgi apparatus and endosomes^23^. Ligand-activated TLR4 dimerizes and is recruited to PtdIns(4,5)P_2_-enriched membrane regions, such as those described in macrophages and epithelial cells^24^,^25^. The ability of cell membranes to present lipid-enriched sites remains unclear. Some studies on the function of the actin filament binding protein MARCKS suggest that membrane proteins modulate the heterogeneous distribution of PIs in membranes. It has been proposed that the binding of MARCKS to PtdIns(4,5)P_2_ limits the lateral diffusion of the PI, facilitating its accumulation at specific sites^26^. Syntaxin-1A clustering at the plasma membrane of PC12 cells is mediated by PtdIns(4,5)P_2_-enriched regions independently of the presence of other negatively charged lipids, such as phosphatidylserine, through electrostatic interactions with its basic residues^27^. It is possible that TIRAP clusters at the plasma membrane using a similar mechanism, independent of the presence of an activated TLR. Thus, TIRAP provides diversified lipid-enriched sites for TLR-mediated signaling. PtdIns(4,5)P_2_-dependent, but phosphatidylserine-independent, TIRAP signaling occurs through TLR4 activation, whereas PtdIns(3)P-dependent TIRAP signaling takes place through endosomal TLR9 activation^11^. In either case, association of TIRAP to these PIs is necessary to recruit MyD88 to PI-enriched membrane regions for the initiation of TLR-mediated signaling pathways.

The N-terminal PBM of TIRAP transitions from a disordered to an ordered structure in the presence of monodispersed PIs and neutral micelles. Common mechanisms are apparent by comparing TIRAP PBM with the Epsin ENTH domain^28^ and the gelsolin PtdIns(4,5)P_2_-binding motif^29^. In these cases, specific phosphate recognition involves a conserved set of tandem basic and nonpolar residues, conformational changes appear to accompany PtdIns(4,5)P_2_ binding, and an increase in their helical content by the presence of lipid is observed. Likewise, a bacterial PI-binding domain (BPD) of the Type III effectors folds upon PtdIns(4,5)P_2_ binding^30^. In the case of the ENTH domain, association with PIs induces the formation of an amphipathic helix, with conserved basic residues contacting the lipid, whereas the hydrophobic outer surface of the helix required for membrane insertion and further changes in membrane curvature^28^,^31^. However, unlike Epsin ENTH, most of TIRAP PBM PtdIns(4,5)P_2_-binding residues are outside of the helix and PIs increase the order in the motif but do not contribute to further membrane penetration of the helix.

Regulation of TIRAP function by multiple post-translational modifications is evident. Caspase-1 removes the helix αE in the TIR domain, rendering the protein unable to interact with MyD88^32^. The Btk-mediated phosphorylation on TIRAP residues Tyr86 and Tyr106, located at its TIR domain, are solvent-exposed and predicted to be localized near the MyD88 binding site^8^. Phosphoregulation of TIRAP on Thr28 by IRAK-1 and −4 is known to promote TIRAP Lys48-linked ubiquitination followed by degradation^16^. Interestingly, Thr28 is located within the PBM of TIRAP. Consequently, we also investigated the role of phosphorylation in TIRAP by the use of a synthetic phosphorylated peptide representing PBM (TIRAP PBM Thr28-P). Binding of TIRAP PBM Thr28-P to PtdIns(4,5)P_2_-embedded liposomes was reduced. Since Thr28 is not directly involved in PI binding, it is likely that it exhibits a structural role as evidenced by its location at the end of the central helix of TIRAP PBM, the orientation of its side chain in the DPC-embedded solution structure, and its interactions with paramagnetic agents. Indeed, Thr28 phosphorylation in TIRAP PBM shows altered secondary structure, with CD minima traces close to 200 nm, indicative of an increment of its random coil content. The use of TIRAP PBM T28D as an approximation of a phosphomimic gave us similar structural and functional results. The significance of phosphorylation in modulating PI binding to TIRAP is highlighted by the demonstration that a TIRAP T28A mutant retains the ability to bind PIs and to localize to cell membranes. Previous reports also showed that phosphorylation on protein domains block PI binding. For example, PKCζ-mediated phosphorylation of the PH domain of the protein kinase B at Thr34 inhibits binding to PtdIns(3,4,5)P_3_, impairing protein kinase B activation by insulin^33^. Structural analysis of the PH domain shows that Thr34 is located at the end of the third β-strand and a few amino acids outside of the PtdIns(3,4,5)P_3_ binding site^34^. As we show for TIRAP PBM, phosphorylation is proposed to induce a conformational change in the protein or reduce the affinity for the PI due to the charge repulsions promoted by the addition of the phosphate group. Likewise, phosphorylation of the PROPPIN family member Atg18, at two distinct loop sites at blades 6 and 7, inhibits binding to PtdIns(3,5)P_2_, thus, preventing Atg18 association to vacuolar membranes^35^. Interestingly, the hydrophobic loop of blade 6 of all PROPPIN members is inserted in membranes^36^, suggesting that, as what likely occurs in TIRAP PBM, the negative charge provided by the phosphate group can prevent membrane insertion. TIRAP phosphorylation and further degradation is thought to prevent LPS over-stimulation, thus, providing tolerance to LPS in situations in which TIRAP-dependent pathways are re-exposed to LPS^3^.

In summary, we demonstrate that TIRAP PBM is a disordered module that undergoes a folding-upon-binding process upon association to PIs. We precisely pinpointed the PI binding residues, which are located at each end of the central helix in TIRAP PBM. TIRAP requires both the head group and the acyl chains of the lipids. We show evidence that phosphorylation of TIRAP in Thr28 leads to a distortion of the helical structure of the PBM, reducing PI binding and membrane localization *in vivo*. These structural changes can then facilitate TIRAP ubiquitination and degradation shortly after TLR activation, providing a mechanism of modulation of innate immune responses.

## Methods

### NMR spectroscopy

NMR experiments were recorded at 25 °C on a Bruker Avance III 600 MHz equipped with a TBI probe (Virginia Tech) and on a cold probe-equipped Bruker 600 MHz (University of Virginia) spectrometers. ^1^H chemical shifts were referenced using sodium 4,4-dimethyl-4-silapentane-1-sulfonate (50 μM), whereas ^15^N- and ^13^C-chemical shifts were referenced indirectly using frequency ratios as described by Wishart and colleagues^37^. Unlabeled, ^15^N and ^13^C, ^15^N TIRAP PBM (0.4–1 mM) were prepared in 10 mM *d*_4_-sodium citrate (pH 6), 50 mM NaCl, 1 mM NaN_3_, 10% D_2_O, and *d*_38_-DPC at a concentration that was ~400-fold of the peptide concentration. Backbone amides were assigned using HNCO, HNCACO, HNCACB, and CACBCONH NMR experiments. Ligand titrations of ^15^N-labeled TIRAP PBM (at a concentration range of 50–100 μM), in the absence and presence of DPC micelles, were performed through ^1^H, ^15^N-HSQC experiments. Ligand-induced resonance perturbations were estimated using the following equation^38^:


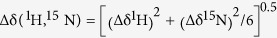


The structure of TIRAP PBM was obtained using the resolution-adapted structural recombination (RASREC) Rosetta. Parameters were selected using the CS-Rosetta toolkit (http://www.csrosetta.org) with Rosetta 3.5 (http://www.rosettacommons.org) assembled with MPI support. Chemical shift information of TIRAP PBM was employed to guide the structure calculation. Rosetta calculations utilized the Janus Supercomputer (UC Boulder), which consists of 1,368 computer nodes, each with two hexcore 2.8 GHz Intel Westmere CPUs, 24 GB RAM, and an 800 TB Lustre filesystem. Calculations were run across 44 nodes, totaling 528 CPUs. The Rosetta calculations yielded 500 RASREC-selected structures of TIRAP PBM, from which twenty structures were selected based on their scores and RMSDs, and converted to Protein Data Bank (PDB) format. The Protein Structure Validation Suite (http://psvs-1_5-dev.nesg.org/) was used to obtain statistics for the twenty lowest energy structures of TIRAP PBM. By using Procheck, the Ramachandran analysis of the twenty lowest-energy conformers of TIRAP PBM identified that 97.5% of the residues are in the most favored regions, 2.5% in the additionally allowed regions, and there were no generously allowed or disallowed regions. All structure images were generated using PyMOL (http://www.pymol.org). The electrostatic potential of TIRAP PBM was calculated using the APBS tool in PyMOL. The structure of DPC-embedded TIRAP PBM has been deposited in the PDB (http://www.rcsb.org) under the accession code 5T7Q and in the BMRB (http://www.bmrb.wisc.edu) under accession code 30170. A Bruker Avance III 600 spectrometer and standard Bruker two dimensional ^1^H-^15^N HSQC-type pulse sequences were used to obtain ^15^N longitudinal (R_1_) and transverse (R_2_) relaxation rates, and ^1^H, ^15^N nuclear Overhauser effects (NOEs). Relaxation delays for the R_1_ experiments were 5, 50, 150, 250, 400, 550, 750, 1200, and 2000 ms. Relaxation delays for the R_2_ experiments were 17, 34, 51, 68, 119, 153, 203, and 254 ms. The recycle delay for R_1_ and R_2_ experiments was 2 s. Spectra with and without ^1^H, ^15^N NOEs were obtained by carrying out experiments with and without 5 s of proton irradiation at the beginning of the pulse sequence. In experiments without ^1^H, ^15^N NOEs, the 5 s proton irradiation was replaced by a 5 s delay. To elucidate the location of TIRAP PBM relative to the micelle surface, two different paramagnetic agents, namely, 16-DSA (Sigma-Aldrich), and MnCl_2_ (J. T. Baker) were used. Due to its poor aqueous solubility, 16-DSA was dissolved in *d*_4_-methanol. Either MnCl_2_ or 16-DSA (up to 1 mM each) was added to TIRAP PBM (0.2 mM) in the NMR buffer containing 50 mM *d*_38_-DPC. The reduction of the resonance intensity of the ^1^H-^15^N HSQC spectra caused by close proximity to the paramagnetic agent was calculated as the ratios of the peak intensities of the spectra in the absence and the presence of the probe. For mapping the location of TIRAP PBM in DPC micelles, in the absence and presence of either c8-PtdIns(4,5)P_2_ or c16-PtdIns(4,5)P_2_, we chose concentrations of paramagnetic agents sufficient to detect at least 75% resonance reduction. NMR spectra were processed with Topspin and NMRPipe^39^ and analyzed by Sparky^40^ and TALOS-N^17^.

### Surface plasmon resonance detection

SPR measurements were performed at room temperature using a BIAcore X100 instrument (GE Healthcare) in 20 mM HEPES (pH 7), and 100 mM NaCl. Dioleoylphosphatidycholine (DOPC) liposomes, with or without 5% PtdIns(4,5)P_2_, were prepared by hydration of a lipid film followed by extrusion using 100-nm membranes (Avanti Polar Lipids). The surface of an L1 sensor chip (GE Healthcare) was preconditioned by injecting 40 mM N-octyl-β-D-glucopyranoside at a flow rate of 5 μl/min. The first flow cell of the sensor chip was used as a control surface (DOPC liposomes), whereas the second flow cell was employed as the active surface (DOPC/PtdIns(4,5)P_2_ liposomes). Approximately 6,000 response units (RUs) of liposomes were immobilized on the surface of the preconditioned L1 sensor chip. Unbound liposomes were washed away with 30 μl of 10 mM NaOH at a flow rate of 30 μl/min. Nonspecific binding sites at the sensor chip surface were then blocked by the injection of 25 μl of 0.1 mg/ml of fatty acid-free BSA (Sigma) at a flow rate of 5 μl/min. A range of concentrations of GST-TIRAP or TIRAP PBM was prepared in the same buffer and injected on both flow cell surfaces at a flow rate of 30 μl/min. Association and dissociation times for each protein injection were set at 120 and 600 s, respectively. The remaining bound protein was washed away by the injection of 5 μl of 70 mM NaOH. The sensor chip surface was regenerated using 40 mM N-octyl-β-D-glucopyranoside. Equilibrium *K*_D_ were estimated by plotting the RUmax levels at the end of the injection *versus* the concentration of the injected peptide and fitted to the steady state affinity using the BIAevaluation software (version 2.0).

### Circular dichroism

Peptide far-UV CD spectra were recorded at room temperature using a Jasco J-815 spectropolarimeter. Five-accumulated scans from 260 to 190 nm were collected on 150-μl volume containing 20 μM TIRAP PBM and mutants in 5 mM Tris-HCl (pH 7) and 40 mM KF in the absence or presence of 20 mM DPC (Anatrace) and/or the indicated ligand and held in a 1-mm path length cell.

### Lipid-protein overlay assay

PtdIns(4,5)P_2_ and PtdIns(3)P (Echelon) membrane strips were prepared by immobilizing 1 μl of the indicated amount of PI dissolved in chloroform/methanol/water (65:35:8) onto Hybond-C extra membranes (GE Healthcare) and dried for 1 h at room temperature. At this point, PI-immobilized membranes were protected from light during the assay. Membrane strips containing the corresponding PI were blocked with 3% (w/v) fatty-acid-free BSA (Sigma) in 20 mM Tris-HCl (pH 8.0), 150 mM NaCl, and 0.1% Tween-20 for 1 h at room temperature. Then, membranes were incubated with the recombinant GST fusion proteins (1 μg/mL) in the same buffer overnight at 4 °C. Following four washes with the same buffer without fatty-acid-free BSA, bound proteins were first probed with rabbit anti-GST antibodies (Santa Cruz Biotechnology) and later with donkey anti-rabbit-horseradish peroxidase antibodies (GE Healthcare). Protein binding was detected using Supersignal West Pico chemiluminescent reagent (Pierce).

### Mammalian cell cultures and immunofluorescence analysis

HEK293 cells (ATCC) were grown in Dulbecco’s minimal essential medium supplemented with 10% (v/v) heat-inactivated fetal BSA (Sigma) in a 37 °C humidified incubator supplemented with 5% CO_2_. Cells were split every 3 d and maintained at about 50% confluency. Transient transfection of TIRAP and mutants, cloned into a pCS2+ EGFP plasmid, were performed using the lipofectamine transfection reagent (Invitrogen) by combining 1 μg DNA and 2.5 μl lipofectamine in OptiMEM reduced serum medium (Invitrogen) per 2 × 10^5^ cells. Cells grown on grid glass coverslips were fixed and permeabilized in 3.7% formaldehyde, 0.1% Triton X-100, and phosphate buffered saline (PBS) for 7 min at room temperature, blocked with 1% BSA (Sigma) in PBS, and incubated overnight at 4 °C. 4′,6-Diamidino-2-Phenylindole (DAPI) (100 ng/ml) and rhodamine phalloidin (5 U/ml) were added to cells to monitor nuclei and actin stress fibers, respectively. Coverslips were mounted onto glass slides with Prolong Gold Antifade Mountant. Images were analyzed with a Zeiss LSM 880 confocal microscope and analyzed using the ZEN 2 software.

## Additional Information

**How to cite this article**: Zhao, X. *et al*. Membrane targeting of TIRAP is negatively regulated by phosphorylation in its phosphoinositide-binding motif. *Sci. Rep.* **7**, 43043; doi: 10.1038/srep43043 (2017).

**Publisher's note:** Springer Nature remains neutral with regard to jurisdictional claims in published maps and institutional affiliations.

## Supplementary Material

Supplementary Information
