# Supplementary material for: Membrane targeting of TIRAP is negatively regulated by phosphorylation in its phosphoinositide-binding motif
Source: Sci Rep. 2017 Feb 22;7:43043. doi: 10.1038/srep43043 (PMC5320522; doi:10.1038/srep43043)
Supplement: Supplementary Information [file srep43043-s1.pdf]

SUPPLEMENTARY INFORMATION

Membrane targeting of TIRAP is negatively regulated by phosphorylation in its phosphoinositide-binding motif

Xiaolin Zhao [1], Wen Xiong [1], Shuyan Xiao [2], Tuo-Xian Tang [1], Jeffrey F. Ellena [3], Geoffrey S. Armstrong [4], Carla V. Finkielstein [5], and Daniel G. S. Capelluto [1,*]

[1] Protein Signaling Domains Laboratory, Department of Biological Sciences, Biocomplexity Institute, and Center for Soft Matter and Biological Physics, Virginia Tech, Blacksburg VA, 24061, USA; [2] School of Materials and Metallurgy, Inner Mongolia University of Science and Technology, P. R. China; [3] Biomolecular Magnetic Resonance Facility, University of Virginia, Charlottesville VA, 22904, USA; [4] Departments of Chemistry and Biochemistry, University of Colorado at Boulder, Boulder CO, 80309, USA. [5] Integrated Cellular Responses Laboratory, Department of Biological Sciences, Biocomplexity Institute, Virginia Tech, Blacksburg VA, 24061, USA.

* Correspondence: capellut@vt.edu (D.G.S.C.)

Methods

Cloning and protein expression and purification

Human TIRAP and TIRAP PBM cDNAs (residues 15-35) were cloned into a pGEX4T1 vector. Recombinant proteins were expressed in *Escherichia coli* (Rosetta strain) cells. Site-directed mutagenesis was introduced in TIRAP and TIRAP PBM constructs using the Quick Change method. Bacterial cells were grown in LB medium at 37°C until they reached an OD_{600} of 0.8 and then recombinant GST-fusion proteins were expressed by the addition of 1 mM isopropyl β-D-thiogalactopyranoside for 4 h at 25°C. ^{15}N-labeled or ^{13}C, ^{15}N-labeled TIRAP PBM was produced in minimal media containing ^{15}N ammonium chloride or both ^{15}N ammonium chloride and U-^{13}C glucose as the nitrogen and carbon sources, respectively. Bacterial pellets expressing GST fusion proteins were purified as we previously reported [1]. GST fusion proteins were eluted with washing buffer containing 100 mM reduced glutathione. TIRAP PBM Thr28-P was synthetically produced at the Tufts University Core Facility (Boston, MS). Protein concentrations were measured using the bicinchoninic acid protein assay reagent.

Table S1. NMR and refinement statistics for TIRAP PBM. NMR structural statistics for the lowest energy conformers of TIRAP PBM using the Protein Structure Validation Suite.

**NMR Dihedral Constraints**	
Dihedral angle restraints	
Total	
ϕ	28
ψ	28
**Structure statistics**	
Dihedral angle constraints (º)	0.3 ± 0.2
Max. dihedral angle violation (º)	7.3 ± 3.8
Deviations from idealized geometry	
Bond lengths (Å)	0.007
Bond angles (º)	0.4
Average pairwise r.m.s. deviation (Å)[a]	
Protein	
Heavy	0.6
Backbone	0.3

[a] Pairwise backbone and heavy-atom r.m.s. deviations were obtained by superimposing residues 15-35 of TIRAP PBM among 20 lowest energy refined structures.

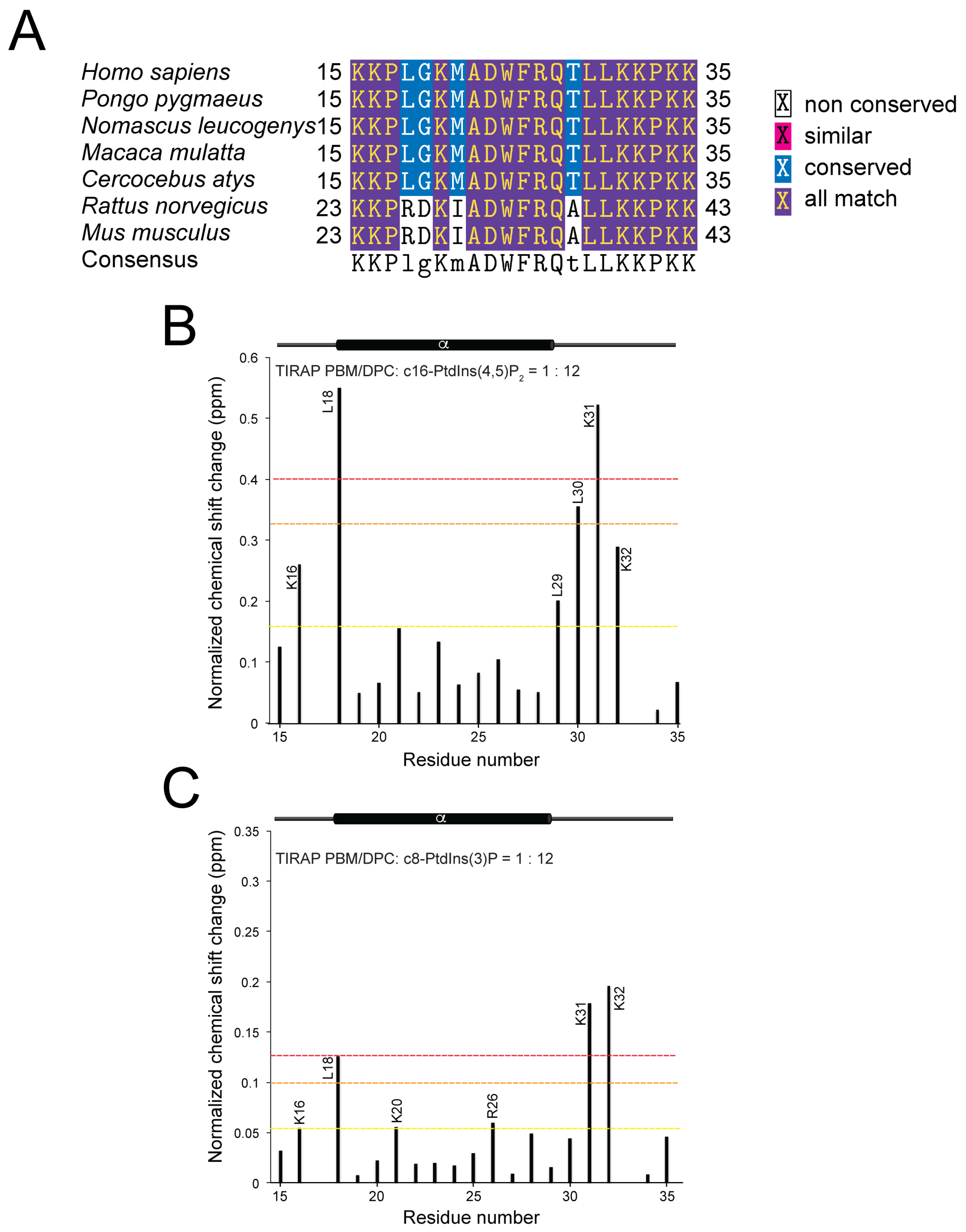

Figure S1. (**A**) Sequence alignment of the TIRAP PBM region from representative mammals. Degree of conservation of amino acids is indicated by the color code on the right. TIRAP PBM homologs include proteins from *Homo sapiens* (GI: 21619620), *Pongo pygmaeus* (GI: 194239513), *Nomascus leucogenys* (GI: 332208648), *Macaca mulatta* (GI: 383415605), *Cercocebus atys* (GI: 795563954), *Rattus norvegicus* (GI: 293349108), and *Mus musculus* (GI: 295390061). Consensus sequences are indicated at the bottom of the alignments. The sequences

were aligned using ClustalW [2]. (**B-C**) Histograms representing the chemical shift perturbations of DPC-embedded TIRAP PBM upon c16-PtdIns(4,5)P_2 (**B**) and c8-PtdIns(3)P (**C**) binding. The colored dashed lines represent significant chemical shift changes: red ($\Delta\delta_{average}$ + 1.5 x standard deviation) > orange ($\Delta\delta_{average}$ + 1 x standard deviation) > yellow ($\Delta\delta_{average}$). Secondary structure of TIRAP PBM is displayed at the top of the histograms.

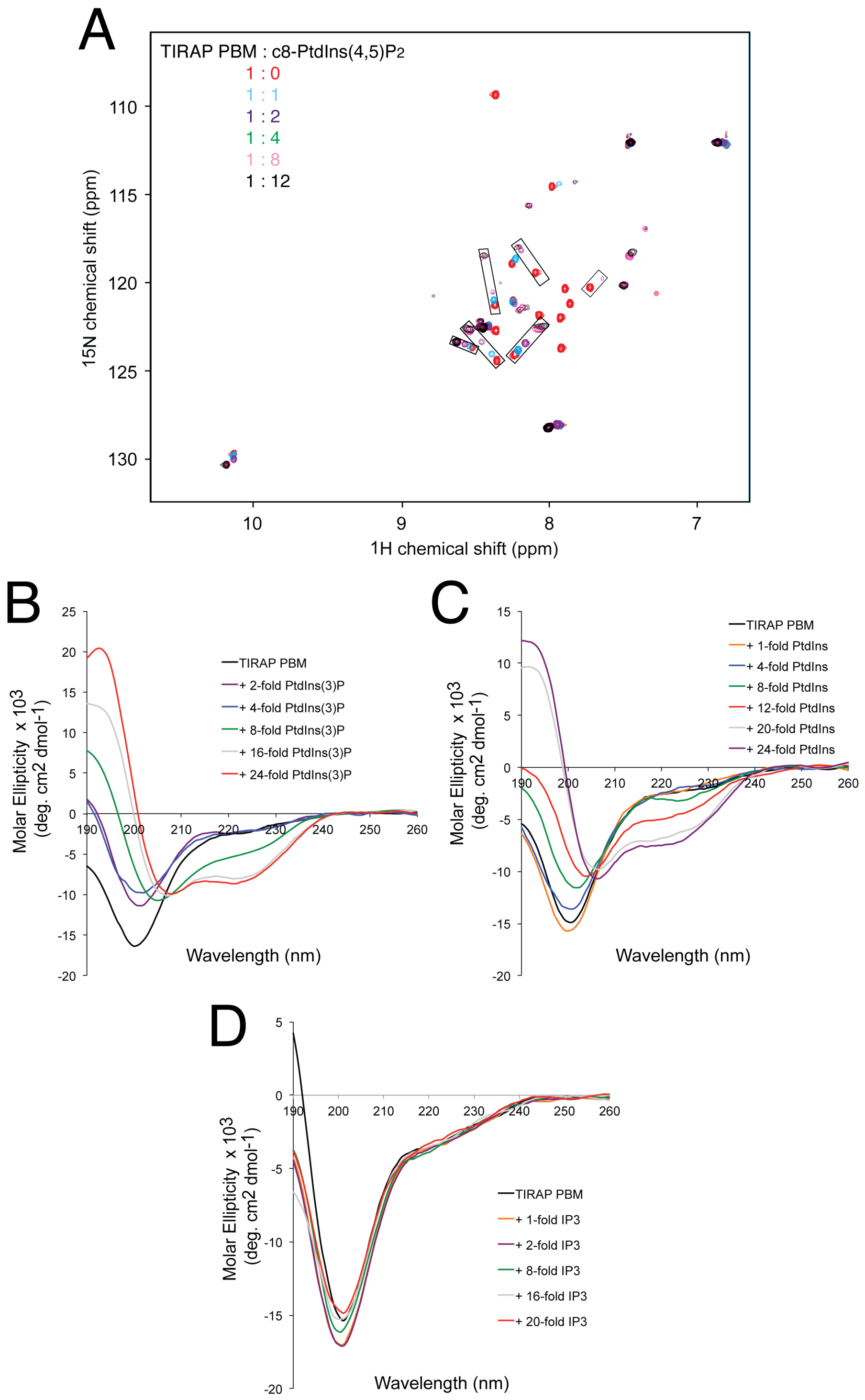

Figure S2. Phosphoinositides induce drastic conformational changes in TIRAP PBM. (**A**) Overlay of $^{1}H,^{15}N$-HSQC spectra of TIRAP PBM in the absence and presence of increasing concentrations of c8-PtdIns(4,5)P_2. (**B-D**) Far-UV circular dichroism spectra of TIRAP PBM in the absence and presence of the indicated molar ratios with c8-PtdIns(3)P (**B**), c8-PtdIns (**C**), and IP_3 (**D**).

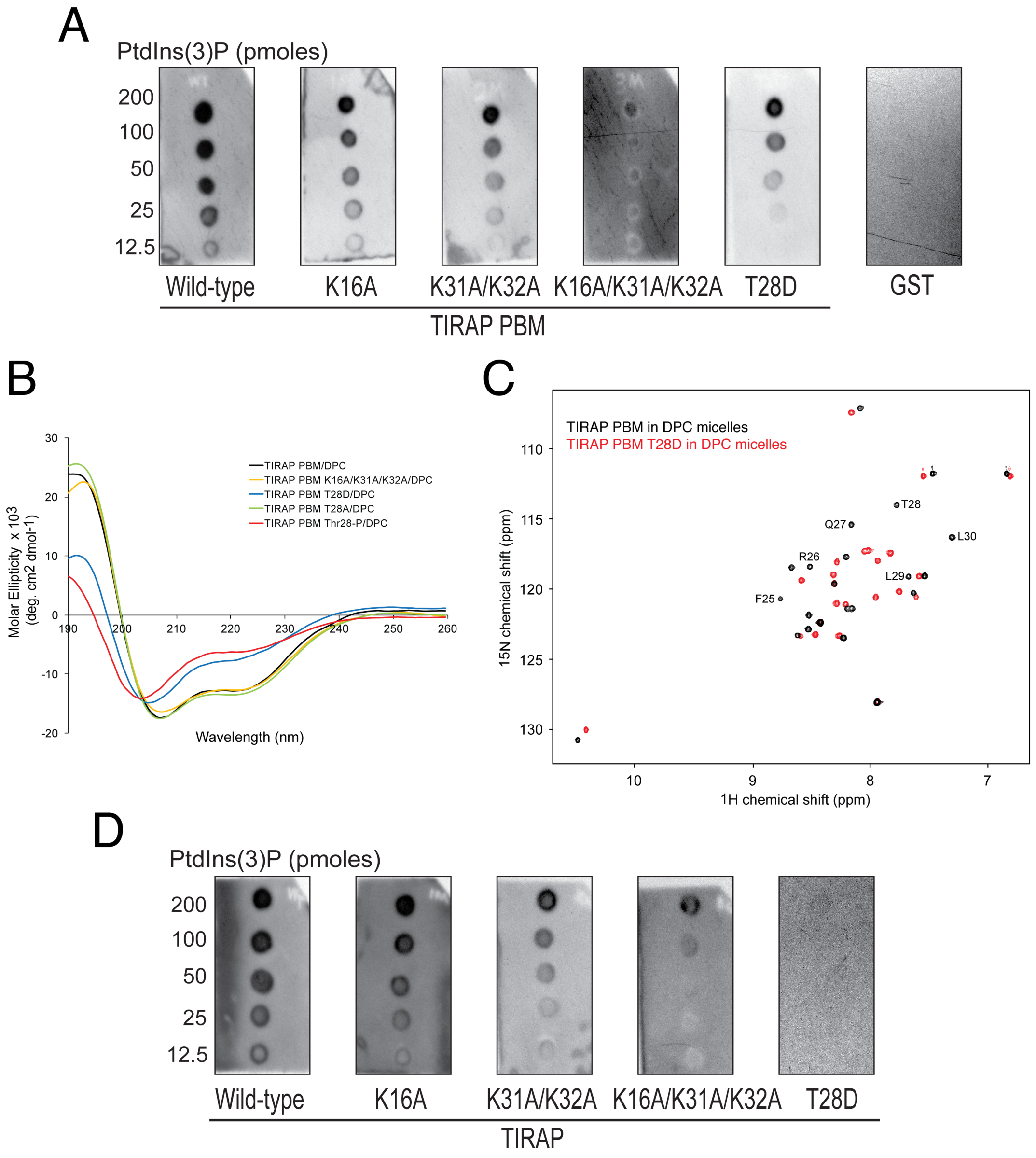

Figure S3. Identification of NMR-based TIRAP residues for PtdIns(3)P binding and the functional and structural role of the modified Thr28 residue. (**A**) Lipid-protein overlay assay of GST-TIRAP PBM and the indicated mutants with immobilized c16-PtdIns(3)P. GST was employed as a negative control. (**B**) Far-UV circular dichroism spectra of DPC-embedded TIRAP PBM and the indicated mutants. (**C**) Overlay of the HSQC spectra of TIRAP PBM (black) and TIRAP PBM T28D (red). Resonances of the TIRAP PBM residues around Thr28 are labeled. Note the loss of dispersion of TIRAP PBM T28D resonances. (**D**) Lipid-protein overlay assay of GST-TIRAP and the indicated mutants with immobilized c16-PtdIns(3)P.

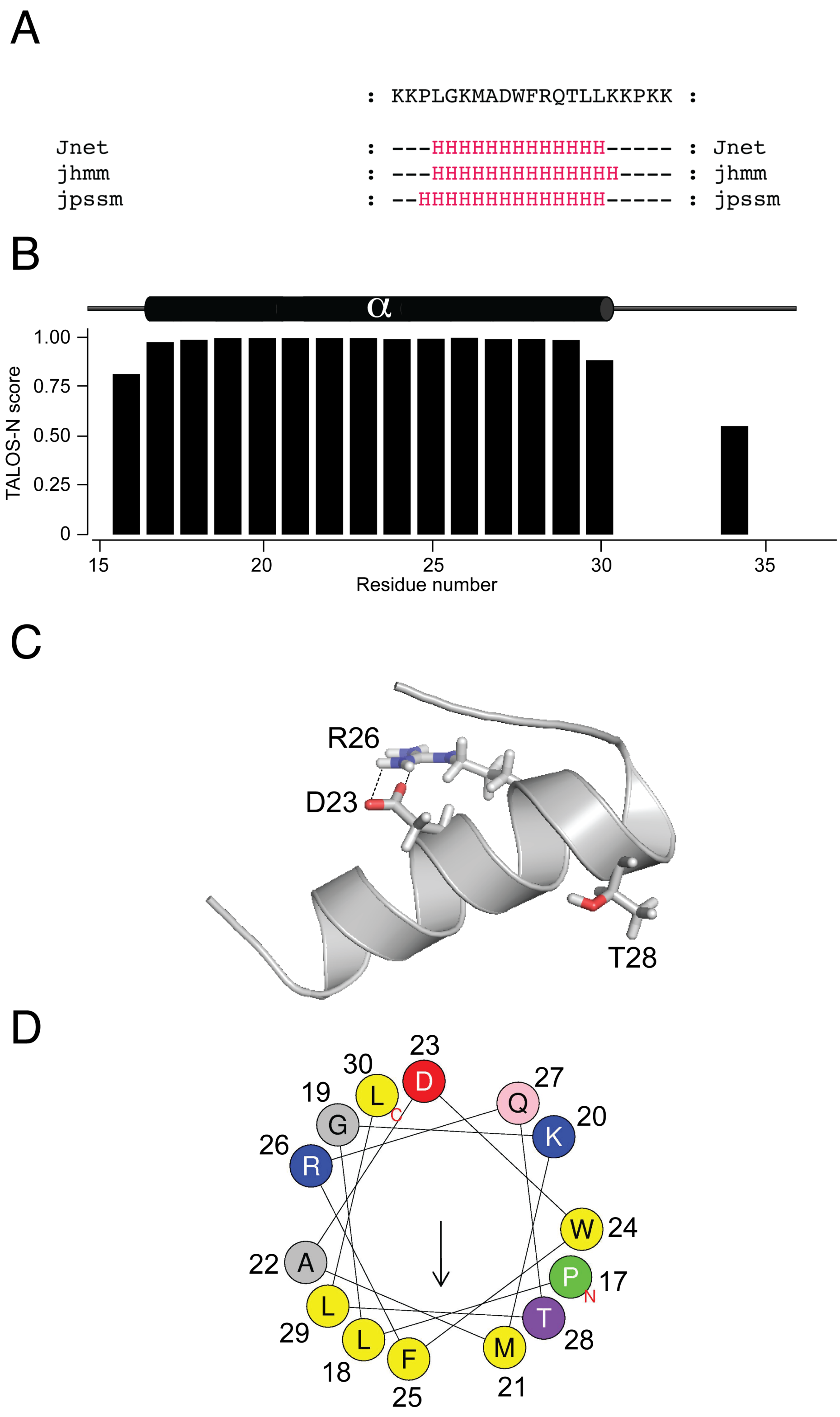

Figure S4. (**A**) Prediction of the secondary structural content of TIRAP PBM using Jpred4 (http://www.compbio.dundee.ac.uk/jpred/). (**B**) Prediction of the secondary structure of TIRAP PBM, using backbone chemical shifts, as determined by TALOS-N. (**C**) Ribbon structure of

TIRAP PBM displaying the location of Asp23, Arg26, and Thr28. Due to their close proximity, Asp23 may form a salt bridge with Asp26. (**D**) Helical wheel projection of TIRAP PBM calculated by using HeliQuest (http://heliquest.ipmc.cnrs.fr/cgi-bin/ComputParamsV2.py). Residues are color-coded based on their chemical properties. The arrow represents the relative hydrophobic moment of the TIRAP PBM helix relative to a perfectly amphipathic peptide.

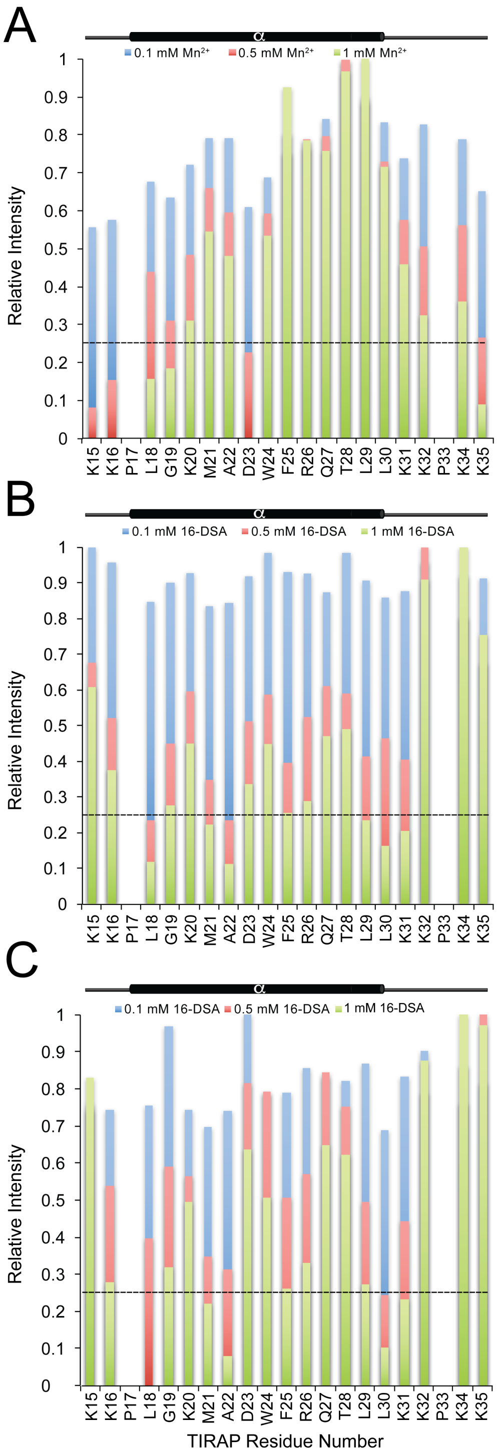

Figure S5. **Probing the insertion of TIRAP PBM in PtdIns(4,5)P_2-embdded DPC micelles.** (**A-B**) c8-PtdIns(4,5)P_2-dependent paramagnetic relaxation enhancement of the backbone amide groups of TIRAP PBM induced by Mn^{2+} (**A**) and 16-DSA (**B**) at the indicated concentrations. (**C**) Same as (**B**) but DPC micelles contained c16- PtdIns(4,5)P_2.

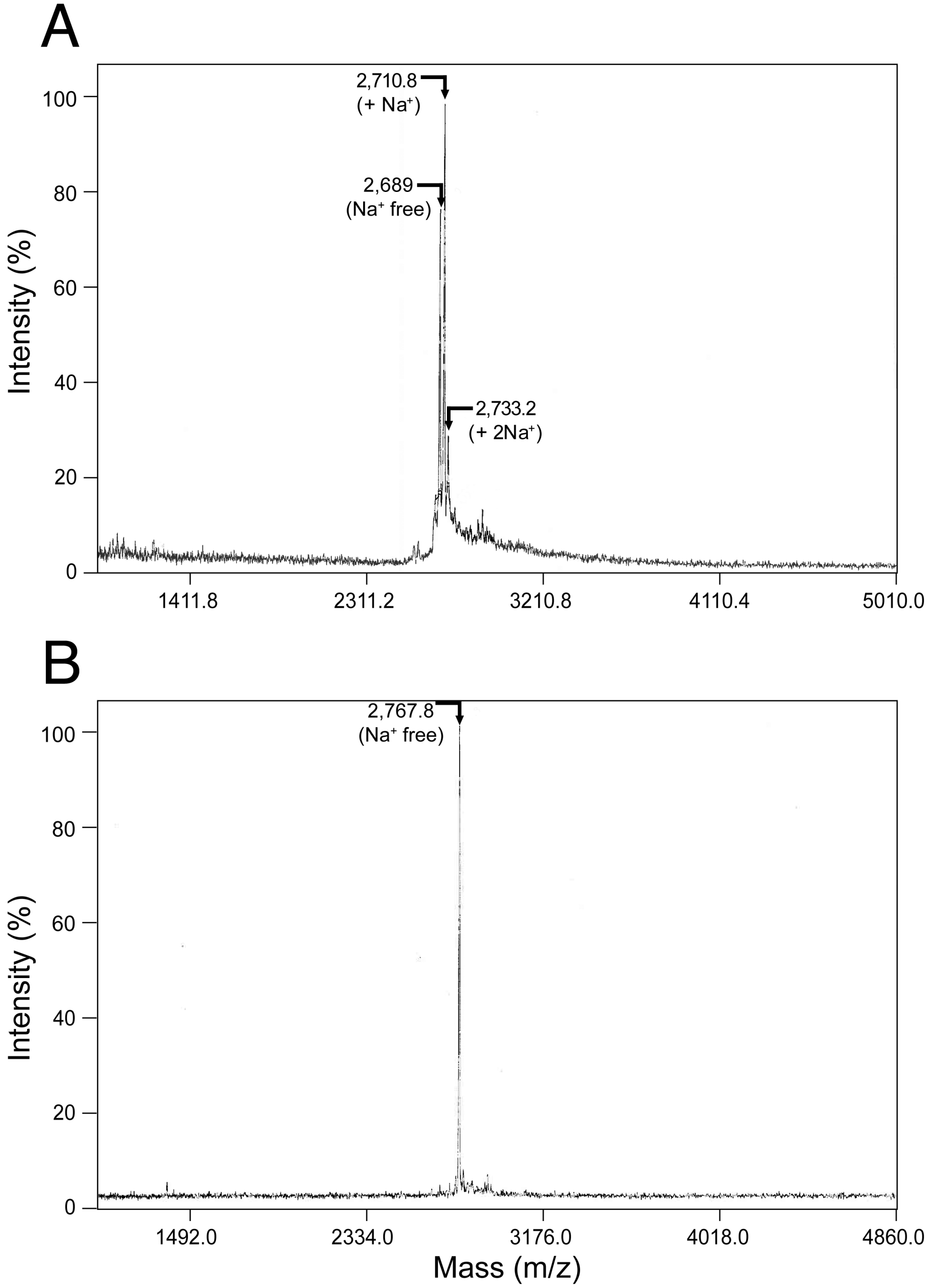

Figure S6. MALDI-TOF data demonstrating the difference in molecular mass of TIRAP PBM (**A**) and TIRAP PBM Thr28-P (**B**). As TIRAP PBM is a recombinant peptide, salt adducts are also present in the sample.

A

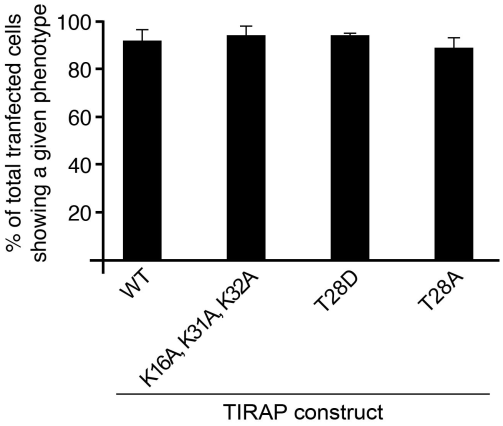

B

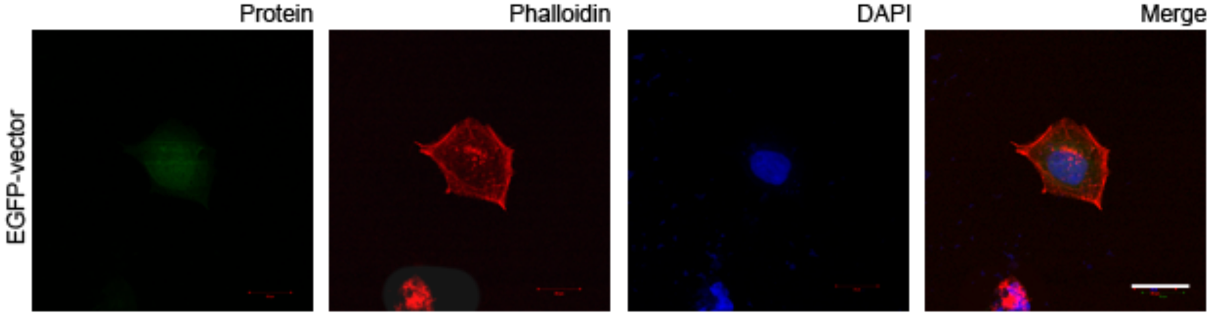

Figure S7. (**A**) Quantification of the immunofluorescence images for a given phenotype. Data represent the quantification of 100 transfected cells per construct from three independent experiments. (**B**) Immunofluorescence control analysis for pCS2+EFGP vector expression in HEK293 cells. Actin stress fibers (phalloidin) and nuclei (DAPI) are colored red and blue, respectively (Bar, 20 μm).

Supplementary References

1. Xiao, S., Zhao, X., Finkielstein, C.V. & Capelluto, D.G. A rapid procedure to isolate isotopically labeled peptides for NMR studies: application to the Disabled-2 sulfatide-binding motif. *J Pept Sci* **20**, 216-22 (2014).

2. Thompson, J.D., Gibson, T.J. & Higgins, D.G. Multiple sequence alignment using ClustalW and ClustalX. *Curr Protoc Bioinformatics* **Chapter 2**, Unit 2 3 (2002).
